# Miliary Tuberculosis Presenting With Meningitis in a Patient Treated With Mycophenolate for Lupus Nephritis: Challenges in Diagnosis and Review of the Literature

**DOI:** 10.1177/2324709618770226

**Published:** 2018-04-18

**Authors:** Precious Macauley, Mark Rapp, Sarah Park, Olaoluwatomi Lamikanra, Pratibha Sharma, Michael Marcelin, Kavita Sharma

**Affiliations:** 1 Maimonides Medical Center, Brooklyn, NY, USA; 2 Yeshiva University Albert Einstein College of Medicine, Bronx, NY, USA

**Keywords:** *Mycobacterium tuberculosis*, lupus nephritis, immunosuppression, mycophenolate mofetil, miliary tuberculosis

## Abstract

Tuberculosis is one of the top 10 causes of death worldwide according to the World Health Organization. Central nervous system involvement is usually the least common presentation of tuberculosis occurring in about 1% of all cases but yet can have very devastating outcomes. Lupus nephritis is one of the most common complications of systemic lupus erythematosus with up to two thirds of patients presenting with some degree of renal dysfunction. The mainstay of treatment is glucocorticoids; however, to sustain remission, steroid sparing agents such as cyclophosphamide, azathioprine and mycophenolate mofetil are used. Such patients, in addition to their baseline dysfunctional immune system, have a heightened risk of infections due to these drugs. In this article, we present a young woman who had recently been started on mycophenolate mofetil for control of class V lupus nephritis who presented with headaches, sinus pressure, and fevers. She had a protracted course of hospitalization as she failed to improve clinically and to respond to conventional therapy for acute bacterial sinusitis and meningitis. She was empirically started on antitubercular therapy 9 days after hospitalization. The diagnosis was not confirmed until day 18, the day results of cerebrospinal fluid acid-fast *bacillus* culture was reported. This case is reported to highlight the challenges in diagnosing *Mycobacterium tuberculosis* infection in an immunocompromised state and to demonstrate that its presentation can mimic numerous other conditions. Clinicians must maintain a high index of suspicion of *Mycobacterium tuberculosis* infection in such patients who present with nonspecific or unexplainable symptoms.

## Introduction

Tuberculosis (TB) is one of the top 10 causes of death worldwide according to the World Health Organization. In 2015, 10.4 million people became ill with a subsequent death toll of 1.8 million. Sixty-one percent of TB cases occur in Asia and 26% in occur in Africa.^1^ TB risk factors include advanced age, HIV infection, malnutrition, alcoholism, and other immunocompromised states.^2,3^ It is well known that patients with systemic lupus erythematosus (SLE) have a dysfunction of both innate and adaptive immune systems, which increases their risk of infections. This risk is further elevated by treatment with immunosuppressive agents.^3^ Evidence suggests that TB may be more prevalent among patients with SLE than within the general population. This increased prevalence is particularly apparent in areas where TB is endemic: with extrapulmonary TB being more common than pulmonary TB, this creates a challenge and delays diagnosis.^3-5^ The least common presentation of TB, occurring in approximately 1% of all cases, is central nervous system (CNS) involvement. Although it is rare, this presentation can have devastating outcomes.^2^

Lupus nephritis (LN) is one of the most common complications of SLE, with up to two thirds of patients presenting with some degree of renal dysfunction.^6,7^ Corticosteroids remain the mainstay of treatment of SLE and LN; however, while they are effective in controlling flares, the effect of corticosteroids on the long-term outcome of patients has not been proven. Maintenance of LN remission is achieved by one or a combination of the following agents: cyclophosphamide, azathioprine, mycophenolate mofetil (MMF), calcineurin inhibitors, and biologic agents.^6,7^ We present the case of a patient with LN, being treated with MMF who develops miliary TB.

## Case Presentation

**Figure 1.**
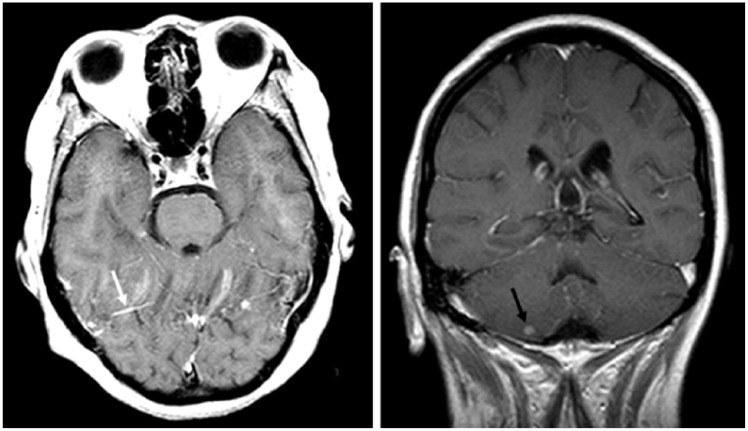
(Left) Axial contrast enhanced flair showing leptomeningeal enhancement in folia of the cerebellum (white arrow). (Right) Coronal contrast enhanced flair showing 0.5 cm nodule in the right inferior cerebellum (black arrow).

A 40-year-old Nepali female with a history of class V LN presented with 4 days of fevers, sinus pressure, chills, rigors, and an occipital headache. She had no cough, weight loss, night sweats, or sick contacts nor had she recently travelled. The patient’s medications included prednisone (20 mg daily for many years) and MMF 500 mg 3 times daily, which was started approximately 2 months prior to presentation. On presentation she was febrile to 103°F; complete blood count revealed no elevation in white blood cell counts but demonstrated neutrophilia at 87%. Computed tomography (CT) showed paranasal sinus inflammatory changes with partial opacification of the right ethmoid sinus and mucosal thickening of the bilateral ethmoid and right sphenoid sinuses. The patient was admitted for acute bacterial sinusitis and treated initially with ampicillin followed by the addition of doxycycline for community-acquired methicillin-resistant *Staphylococcus aureus* coverage. On the third day, sinus pressure had abated; however, the patient had persistent spiking fevers with occipital headache. Infectious Disease service was consulted on day 4 for evaluation and recommendations regarding persistent fever spikes in an immunocompromised patient. At this point, the patient had no sinus tenderness, neck stiffness, or nuchal rigidity. A lumbar puncture was performed and results suggested a partially treated bacterial meningitis (see Table 1). A lupus flare was ruled out as dsDNA and complement levels were within normal limits (see Table 2). On the same day (day 4), the patient was started on a 5-day course of vancomycin, cefepime, ampicillin, and acyclovir pending complete results of the cerebrospinal fluid (CSF) analysis. Magnetic resonance imaging (MRI) of the brain obtained due to persistent headaches revealed leptomeningeal enhancement in the folia of the cerebellum (Figure 1). The clinical picture of spiking fevers in an immunosuppressed patient, together with the CSF and imaging findings, raised suspicion of CNS TB or an opportunistic fungal infection.

**Table 1.** CSF Results.

	Appearance	Glucose	Protein	WBCs	RBCs
First CSF, day 4 of admission	Colorless/clear	<10	137	426 (50% N, 46% L, 5% M)	200
Second CSF, day 9 of admission	Colorless/clear	<10	169	218 (differential not given but many lymphocytes)	138

Abbreviations: CSF, cerebrospinal fluid; WBCs, white blood cells; RBCs, red blood cells; N, neutrophils; L, lymphocytes; M, monocytes.

**Table 2.** Inpatient Workup.

Infectious	CSF meningitis/encephalitis panel: no detection
	• Panel tests for *Escherichia coli; Haemophilus influenza, Listeria monocytogenes, Neisseria meningitides, Streptococcus agalactiae, Streptococcus pneumoniae*, cytomegalovirus, enterovirus; HSV-1, HSV-2, human herpesvirus 6, parechovirus; varicella zoster virus, and *Cryptococcus neoformans*
	*Bacterial*:
	• Serum Quantiferon-TB Gold Test: negative
	• Procalcitonin: <0.05 ng/mL (0.00-0.04 ng/mL)
	• CSF: VRDL negative
	○ Lyme borderline
	○ CSF AFB smear—positive after 18 days
	○ CSF cultures: (CSF 1) AFB detected in broth on day 18
	○ Adenosine deaminase: 1.8 U/L (normal <7 U/L)
	• Sputum AFB smear: negative until post discharge
	• Bronchial lavage AFB smear: positive post discharge
	• Urine cultures ×4—no growth
	• Blood cultures ×10—no growth
	• Echocardiogram: no vegetations
	*Fungal*:
	• CSF: Cryptococcus antigen A and B negative
	• CSF fungal cultures ×2—negative
	• Serum 1,3-B-D-glucan assay—negative
	• Bronchial lavage fungal—no growth
	• *Histoplasma capsulatum* and *Coccidiodes immitis* antibody negative, *Aspergillus* assay negative
	*Viral*:
	• CSF: parvovirus antibody B19 IgM and IgG negative
	• HSV negative; JCV antibody positive
	• EBV IgG: positive, IgM negative
	• HIV1/2: negative by enzyme immunoassay
Neoplastic	CSF cytology negative for malignant cells
Rheumatologic	Complement C3, CH50 and dsDNA—normal
Imaging	Day 0: CT without contrast of head—suggestive of sinusitis and mucositis Day 8: MRI brain—small areas of leptomeningeal enhancement Day 10: CT chest, abdomen, and pelvis: no intraabdominal abscess, multiple tiny nodules scattered in bilateral lungs Day 22: MRI brain—stable mild leptomeningeal enhancement

Abbreviation: CSF, cerebrospinal fluid; HSV, herpes simplex virus; AFB, acid-fast bacilli; IgM, immunoglobulin M; IgG, immunoglobulin G; EBV, Epstein-Barr virus; CT, computed tomography; MRI, magnetic resonance imaging.

**Figure 2.**
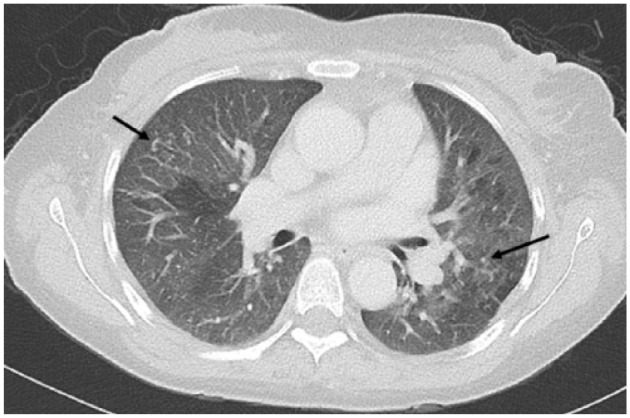
Computed tomography angiography at current presentation. Black arrows indicate bilateral scattered nodules consistent with miliary tuberculosis.

**Figure 3.**
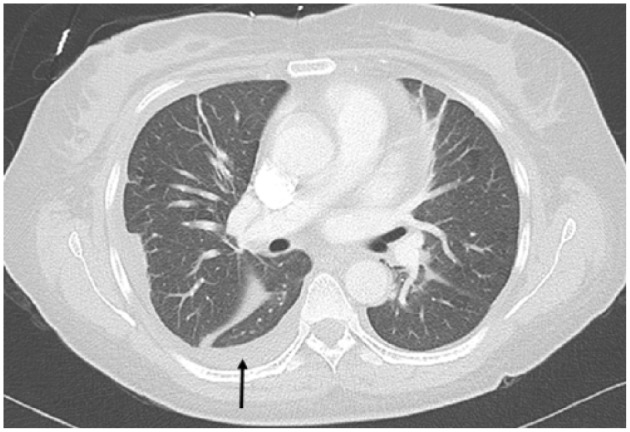
Computed tomography angiography 4 months prior to current presentation. Black arrow showing trace pleural effusion.

On day 9, a second lumbar puncture was performed and sent for analysis. The patient was started on empiric antitubercular therapy with rifampin, isoniazid, pyrazinamide, and ethambutol despite a negative QuantiFERON-TB Gold test (QTF-G), chest radiography showing no evidence of pulmonary involvement, and initially negative sputum acid-fast bacilli (AFB) smears. A CT angiography of the patient’s chest, abdomen, and pelvis revealed multiple tiny nodules scattered in bilateral lung fields (Figure 2), not observed in a CT chest scan performed 4 months earlier (Figure 3).

Over the next few days the patient remained febrile with headaches; CSF and sputum cultures revealed no organism growth and malignant cells were absent in CSF cytology. Broad-spectrum antibiotics to cover bacterial and fungal meningitis were continued. On day 16, bronchoscopy with bronchoalveolar lavage was performed and samples sent for AFB smear, herpes simplex virus polymerase chain reaction and cryptococcal antigen.

The patient slowly became afebrile, with a decrease in the intensity of headaches and restoration of appetite, and on day 18, results from the first sample of CSF was positive for AFB, identified as *Mycobacterium tuberculosis* (MTB). The patient was discharged on day 25 after admission to follow-up with the Department of Health. After the patient’s discharge, sputum, CSF, and bronchial cultures were reported positive for MTB.

## Discussion

SLE patients have an increased risk of infections; TB in particular occurs in SLE patients at a rate of 3.6% to 5%, which is higher than in the general population.^3,8^ Glucocorticoids, the cornerstone of the management of SLE and numerous other autoimmune diseases, exert their effect through suppression of T-lymphocyte-mediated immunity, which takes approximately 21 days to become long lasting.^8^ Studies have shown that glucocorticoid doses below 5 mg per day do not confer an increased risk of developing infections.^8^ In one case series by Vargas et al of 23 SLE patients with CNS infections, 30.4% were diagnosed with MTB and their average daily dose was 28.9 mg of prednisone.^9^ This finding can be corroborated by the retrospective study conducted by Hou et al,^5^ who discovered that of the 19 patients who developed clinical TB only 6 (31.6%) were taking less than 20 mg of prednisone per day, and the average cumulative daily dose of prednisone was 37.7 mg.

The general consensus among major societies is that for class III and class IV LN (the 2 classes with active proliferative glomerulonephritis), therapy consists of steroids in addition to cyclophosphamide or MMF. However, the latter has increasingly been used as first-line therapy for LN due to its better safety profile and lower rates of relapse when compared with cyclophosphamide.^6,10^

MMF is converted in vivo to mycophenolic acid, the active immunosuppressive agent that inhibits the synthesis of guanosine nucleotides, thereby depleting both T- and B-cell population. It is therefore no surprise that infections are a common complication as both humoral and cellular immunity are profoundly suppressed.^11,12^ However, when compared with the older agent cyclophosphamide, MMF causes less bone marrow suppression and hence is associated with less infectious complications.^7,10^ When compared with azathioprine, the other first-line agent in the treatment of LN, studies have not shown a difference in rates of infection.^7,13^ The exact increase in the risk of developing TB in SLE patients being treated with MMF has not yet been clearly investigated, although one case series by Bhattacharya et al showed 2 out of the 5 patients who developed TB were being treated with MMF. One of the patients was diagnosed with pulmonary TB and the other with brain tuberculomas.^4^ Several case reports looking at renal transplant recipients showed reactivation of latent TB once azathioprine was switched to MMF.^11,14,15^ In our patient, we have no tuberculin skin test (TST) to show prior status; however; the CT scan performed 4 months prior to this presentation showed no cavitary lesions, nodules, or opacifications. The pleural effusions were most likely due to the patient’s fluid overload at the time of the CT scan (Figure 3).

The challenges in diagnosing TB stem from the nonspecificity and extrapulmonary manifestations such as joint swelling, arthralgia, unexplained pulmonary infiltrates, dyspnea, fever, and cough. The differential diagnosis can include pleurisy, inflammatory arthritis, and cellulitis.^4,5,16^ The retrospective analysis by Balakrishnan et al^17^ in Mumbai found the average time to diagnosis to be 22.5 days, whereas it took over 60 days to reach a diagnosis according to Hou et al in Taiwan.^5^ In our patient, definitive diagnosis was made 18 days after presentation, although an empiric anti-TB regimen was started on day 8 as clinical suspicion was high.

The TST, which was originally the gold standard in the diagnosis of latent TB, has limitations, which include false positivity in individuals who received the Bacillus Calmette-Guerin (BCG) vaccine and false negativity in those who are chronically immunosuppressed.^18^ Our patient had received the BCG vaccine as a child in Nepal. QTF-G has been used to aid the diagnosis of latent tuberculosis infection as it possesses higher sensitivity than TST and better specificity in the BCG-vaccinated population.^18,19^ The QTF-G test assesses the response to synthetic overlapping peptides such as early secretory antigenic target-6 and culture filtrate protein-10 that are usually present in all MTB strains. These proteins which act as antigens are incubated with whole blood and stimulate the release of interferon-γ, which is measured by enzyme-linked immunosorbent assay.^18,19^ This test is not without its drawbacks; several studies have shown that a large proportion of SLE individuals show an indeterminate result when the QTF-G test is performed. One study by Takeda et al^19^ showed that 32.4% of SLE patients fell in this category. Factors that were associated with indeterminate results included lymphocytopenia and a high SLE disease activity index. Other proposed contributing factors are old age, hypoalbuminemia, and use of immunosuppressive agents. Our patient demonstrated nearly all of these traits, she had low serum albumin (between 1.8 and 2.8 g/dL), lymphocytopenia (percentages of as low as 7.2%), and was on long-term immunosuppressive agents. It is therefore safe to state that neither latent tuberculosis infection nor active TB in SLE patients can be diagnosed using the TST or the QTF-G assay.

Another emerging diagnostic tool is the adenosine deaminase (ADA) activity in CSF. ADA is an enzyme that converts the purine adenosine and deoxyadenosine to inosine and deoxyinosine, which are essential to the differentiation and proliferation of lymphocytes in cell-mediated immunity particularly in T lymphocytes.^20^ This test is mainly used when analyzing pleural fluid when there is a moderate to high suspicion of a tuberculous effusion; however, several studies have begun to utilize this test in the analysis of CSF in similar cases. There have been discrepancies in the cutoff values among these studies; for example, Agarwal et al^20^ found that a cutoff value of 10 IU/L yielded a sensitivity and specificity of 87.5% and 83.3%, respectively, whereas, the study by Raviraj et al^21^ proposed a lower cut-off of 6.65 IU/L with sensitivity and specificity of 85.3 and 84.3%, respectively. Ekermans et al^22^ used an even lower cutoff value of 2 IU/L and yielded sensitivity and specificity values of 85.9% and 77.7%, respectively. They also found that 13 out of 92 TB meningitis cases were missed initially. Our patient had a CSF ADA level of 1.8 IU/L, and our facility had a cutoff value of 7 IU/L, and using this value gives a false negative result and was not useful in ruling out the TB diagnosis.

It is extremely important to make a TB diagnosis in order to prevent the devastating outcomes that could arise as a consequence of the complications of disseminated TB infection particularly when the CNS is involved. TB in the CNS can manifest in 3 categories: subacute, chronic, or intracranial tuberculomas.^23^ The classic presentation is a prodrome of anorexia, low-grade fevers, fatigue, and headaches; not all individuals demonstrate signs of meningeal irritation. Our patient had nonspecific symptoms of headache and facial pain and had no neck stiffness or focal neurological deficits. The complications of CNS TB include cranial nerve palsies, hemiparesis, hemiplegia, and cerebrovascular accidents. A small subset of patients may demonstrate a syndrome of slowly progressive dementia, personality changes, and social withdrawal.^3,23^

## Conclusion

Taken together, the nonspecificity of symptoms, lack of classic radiologic evidence, and indeterminate serologic and laboratory test results that can be encountered in an SLE patient with TB should cause clinicians to maintain a high index of suspicion when a patient presents with nonspecific symptoms of fevers, chills, headache, and myalgias, especially once a lupus flare has been ruled out through the demonstration of low titers of dsDNA and normal levels of complement.
